# Between-Session Homework in Clinical Training and Practice: A Transtheoretical Perspective

**DOI:** 10.32872/cpe.12607

**Published:** 2024-04-26

**Authors:** Truls Ryum, Nikolaos Kazantzis

**Affiliations:** 1 Department of Psychology, Norwegian University of Science and Technology, Trondheim, Norway; 2 Cognitive Behavior Therapy Research Unit, Melbourne, Victoria, Australia; 3 Beck Institute for Cognitive Behavior Therapy and Research, Philadelphia, PA, USA; Department of Psychology, University of Trier, Trier, Germany

**Keywords:** homework, training, practice, trans-theoretical, evidence-based

## Abstract

**Background:**

This paper defines and illustrates the ways in which Between-Session Homework (BSH) may be integrated into clinical work with clients across various treatment approaches. In line with the focus of this special issue, we explore how clinical training and supervision can enhance therapist skills and competence in the use of BSH.

**Method:**

After providing a brief historical overview and an integrative perspective on BSH, along with a review of empirical research supporting its efficacy, we delve into the discussion of BSH as a transtheoretical clinical method with heuristic value across different treatment approaches, such as cognitive-behavioral, psychodynamic, and humanistic-experiential therapies.

**Results:**

There exists diversity in how BSH is incorporated into distinct treatment approaches. Furthermore, we emphasize the significance of therapist skills and competence in utilizing BSH to facilitate client engagement and achieve positive treatment outcomes. Finally, we address how clinical training and supervision contribute to the development of these essential skills and competence.

**Conclusions:**

Our findings highlight three main points: (1) substantial empirical support for the integration of BSH within cognitive-behavioral therapies, (2) the potential of BSH as a promising transtheoretical clinical method, even though research beyond cognitive-behavioral therapies remains limited, and (3) the imperative need for further research into how clinical training and supervision can effectively enhance therapist skills and competence in implementing BSH.

## Highlights

- Between-session homework (BSH) shows promise as a transtheoretical clinical method for clinical practice and training.
- Therapist skills and competence in utilizing BSH are essential to facilitate client engagement with BSH and treatment outcomes.
- Research into how clinical training and supervision can effectively enhance therapist skills and competence in implementing BSH is needed.

## A Brief Historical Overview of Between-Session Homework (BSH)

The utilization of Between-Session Homework (BSH) holds a substantial historical significance within behavioral and cognitive-behavioral therapies (CBT). It was initially introduced as a systematic strategy for promoting and sustaining behavior change in behavior therapy (Shelton & Ackerman, 1974; Shelton & Levy, 1981). As cognitive therapy gained prominence, the significance of between-session assignments in achieving successful outcomes gathered momentum. Notably, Aaron T. Beck and colleagues dedicated a separate chapter to the use of BSH in their seminal clinical guide, "*Cognitive therapy of depression*" (Beck et al., 1979). While there has been a proliferation of training resources (e.g., Beck, 2020) and treatment manuals for specific disorders since then, there has also been a recent shift towards more integrative and transtheoretical models (e.g., Barlow et al., 2017; Barlow et al., 2011). Despite these changes, the importance of BSH remains a defining feature of CBT. This significance is underscored in the recent conceptual model proposed by Kazantzis and Miller (2022). In fact, the literature on homework is most advanced within CBT, with more research supporting its positive impact on outcomes compared to any other treatment component (see reviews in Kazantzis et al., 2018; Ryum et al., 2023a, 2023b).

While BSH is considered a primary driver of change in CBT, it has received relatively less attention within psychodynamic (PDT) and humanistic-experiential therapies (HET). In these approaches, BSH is typically assigned a more supportive role in the therapeutic process and outcome. However, even Freud proposed the incorporation of between-session activities in psychoanalysis (Freud, 1952), and subsequent psychodynamic authors have advocated for integrating BSH into psychodynamic therapy (e.g., McCullough, 2003; Stricker, 2006; Wachtel, 1977). This notion is further supported by surveys of practicing therapists, indicating that BSH is commonly utilized by both cognitive-behavioral and psychodynamic therapists (Fehm & Kazantzis, 2004; Kazantzis & Dattilio, 2010; Kazantzis, Lampropoulos, et al., 2005). Similarly, within humanistic-experiential therapies, the use of BSH has been endorsed (e.g., Brodley, 2006; Greenberg & Warwar, 2006; Warwar & Ellison, 2019), although a comprehensive survey among practicing therapists is lacking to our knowledge. It is worth noting that the *Journal of Psychotherapy Integration*, Vol. 16, No. 2, featured a dedicated special issue on the topic of homework across various psychotherapeutic models.

We note that there has also been substantial interest in the affiliated concept of “intersession experiences/ processes” (Orlinsky & Geller, 1993; Orlinsky et al., 1993), which builds upon psychodynamic- and transtheoretical theories, and refers to the client’s (and therapist’s) spontaneous or intentional processing of psychotherapy between session, including thoughts, feelings, memories, and fantasies about the therapy or the therapist (Orlinsky et al., 1993). These experiences are internalized over the course of therapy, forming affect-laden representations, that influence on the therapeutic process and outcome (e.g., Hartmann et al., 2010; Hartmann et al., 2016; Zeeck & Hartmann, 2005). While both intersession processes and BSH concerns between-session activities, and client’s intersession experiences may relate to BSH, they differ fundamentally in the sense that BSH is planned, targeted, and negotiated between therapist and client through in-session dialogue, whereas intersession processes are not. We therefore refer interested readers to other sources for recent reviews on intersession processes (Gablonski et al., 2023; Stewart & Schröder, 2015). For similar reasons, we avoid further discussion of between-session activities that are primarily client-initiated (e.g., spontaneous implementation of treatment-related content in stressful situations), although such activities would clearly be considered therapeutic (see discussion in Brodley, 2006).

Furthermore, while our primary focus here centers on individual therapy, it is also imperative to acknowledge the enduring interest in the utilization of BSH within the contexts of couples and family therapies. This enduring interest is evidenced through surveys conducted among practicing therapists and their clinical applications (Dattilio et al., 2011; Kazantzis et al., 2023). The pervasive employment of BSH is thus discernible across various psychotherapeutic approaches, to the extent that it has been posited as a potential 'common factor' (Kazantzis & Ronan, 2006). Nonetheless, disparities persist in the manner by which BSH is assimilated into clinical practices across these treatment approaches—an issue we shall expound upon. It is noteworthy to highlight that substantial endeavors have been undertaken to facilitate conciliation between therapeutic paradigms in the conceptualization of BSH as a transtheoretical method (a forthcoming special issue in the *Journal of Clinical Psychology – In Session*, 2024, delves into this matter). Importantly, an enduring interest in the application of BSH endures, both within the framework of Cognitive Behavioral Therapy (CBT) and across other diverse treatment methodologies (Ryum et al., 2023a, 2023b), with mounting empirical support that substantiates BSH as an evidence-based, transtheoretical method of significance for clinical practice and the training of clinical psychologists.

## Between-Session Homework: An Integrative Perspective

Numerous terms and definitions have been proposed to encapsulate the fundamental essence of Between-Session Homework within the context of psychotherapy. These designations, encompassing a range of connotations such as “homework,” “extra-therapy assignment,” and “home practice activities,” among others, do not inherently signify a transformation in the content, nature, or essential essence of BSH. In this paper, we adopt the term “Between-Session Homework,” recognizing the absence of a universally established terminology. Nonetheless, it is imperative to underscore that BSH serves as a conduit for fostering therapeutic advancement and ultimate treatment objectives. A comprehensive generic definition of BSH may be formulated as “activities enabling clients to assimilate information and extrapolate newfound insights from the therapeutic milieu into their everyday life contexts, wherein their challenges transpire.” This definition encompasses both “insight-oriented” elements such as information and awareness, and “action-oriented” components like new learning and skill acquisition, which have been posited as universal mechanisms for change in psychotherapeutic contexts (e.g., McCullough et al., 2003; Nelson et al., 2007; Ryum et al., 2014; Valen et al., 2011). Furthermore, this elucidation underscores the inherent reality that, for each therapeutic hour dedicated, the remaining 23 hours of the day are lived beyond the counseling environment. Thus, it intuitively follows that this time outside the therapy room should be harnessed to expedite the therapeutic process and advance toward ultimate treatment goals (Kazantzis, Deane, et al., 2005).

Diversity characterizes the content and nature of BSH across distinct treatment methodologies. The timing, inclusion, and modality of BSH in psychotherapy necessitate adaptability, both within and across therapeutic approaches. The specific nature of BSH tasks and the procedural dynamics within a therapeutic session with a given client are contingent upon an array of factors, including the unique requirements of the client, the rationale underpinning the chosen therapeutic approach, the client's stage of change (e.g., precontemplation, contemplation, preparation, action, maintenance; Prochaska & Norcross, 2018) and contextual variables. To elaborate further, BSH might encompass activities such as relaxation training, exposure exercises (in vivo, imaginal, interoceptive), activity scheduling, behavioral activation, integration of newly acquired skills (e.g., experimenting with novel interpersonal strategies or modes of interpersonal engagement), behavioral experiments, and the acquisition of information and heightened awareness (e.g., recording automatic thoughts, attending to dreams or emotional responses). BSH can effectively address behaviors and symptoms linked to specific settings or individuals in the client's daily life beyond the therapeutic encounter, as exemplified in various anxiety disorders (e.g., agoraphobia, obsessive-compulsive disorder) or maladaptive interpersonal patterns or self-other relational dynamics (e.g., personality disorders).

Amid this diversity, a central unifying rationale for BSH is the cultivation of learning in various forms (Kazantzis & L’Abate, 2007). BSH serves to inform, consolidate, expand, and reinforce the clinical work transpiring within the therapy session, enabling the continuation of therapeutic progress into the client's daily life where challenges most persist. The emphasis on BSH accentuates the client's active involvement in the therapeutic journey and may foster a sense of agency and responsibility for effecting positive change in their lives (Dobson, 2022; Dobson & Kazantzis, 2023; Strunk, 2022). Inherent to this endeavor is the promotion of a mindset characterized by curiosity, interest in one’s thoughts and emotions, and the cultivation of self-care and self-acceptance.

Diverse perspectives arise when considering the in-session dynamics and therapist behaviors germane to the integration of BSH into psychotherapy. Notably contentious is the question of whether BSH should predominantly emanate from the therapist (Ellis, 1962), be initiated autonomously by the client (Brodley, 2006), or emerge through collaborative negotiation between therapist and client (Kazantzis et al., 2013; Kazantzis et al., 2017). In most cognitive and behavioral therapeutic modalities, the delineation, planning, and review of BSH are explicitly incorporated into the session agenda, ideally arrived at through collaborative empiricism between client and therapist. Homework assignments are meticulously tailored to the individual client, grounded in evidence-based therapeutic strategies and contextual considerations, often accentuating the specificity of the execution of BSH (e.g., how, when, where, duration, frequency, interpersonal involvement), thus augmenting the likelihood of successful outcomes (Hildebrand-Burke et al., 2023; Kazantzis & Miller, 2022).

Conversely, psychodynamic and humanistic-experiential therapies typically do not accord the same explicit prominence to the selection, design, and review of BSH within the session context. These therapeutic paradigms lean historically towards a less specific stance, emphasizing in-session processes such as the client-therapist relationship and the exploration of subjective experiences, emotions, and meaning, in contrast to activities external to the session. Consequently, the introduction of BSH in these approaches is characterized by a more indirect and tentative manner; for instance, a humanistic-experiential therapist might propose BSH as an optional experiment or as a potential avenue for the client's exploration if deemed beneficial (Brodley, 2006), for example, with the use of tasks to increase self-soothing capabilities or the use of a diary to promote emotional awareness. Meanwhile, a psychodynamic therapist might adopt a hypothetical approach to between-session assignments, such as positing, “I wonder what might have transpired had you...”, or even suggest more directively that the client tries out new interpersonal behaviors or ways of relating to others (Dimaggio et al., 2015). It is important to highlight that a CBT therapist might also choose to use similar language and express a level of tentativeness. This approach could be prompted by their ongoing case formulation, suggesting that a straightforward expectation regarding BSH may potentially result in an alliance rupture. For instance, this might occur in situations involving an active abuse/mistrust schema being transferred to the therapist (refer to the discussion in Kazantzis et al., 2017).

As previously alluded to, current trends in the field indicate a burgeoning framework for BSH underscored by integration and assimilation. For instance, BSH may encompass endeavors aimed at heightening client awareness regarding latent thoughts and emotions or promoting self-compassion and self-acceptance (Hayes, 2022). Irrespective of the precise nature of the homework task, it is crucial to underscore that the effective and successful utilization of BSH hinges on the establishment of a reciprocal and collaborative therapeutic relationship, where the therapist's facilitative interpersonal skills are indispensable, and consensus between therapist and client prevails regarding the specific objectives and tasks of the therapeutic process (Kazantzis et al., 2017). These qualities appear to characterize the in-session process of integrating BSH across treatment approaches, as recently demonstrated (Ryum et al., 2024a, 2024b).

In summary, the panorama of BSH in psychotherapy is characterized by a mosaic of perspectives, encompassing diverse terms and definitions, multifaceted modalities, and a rich interplay between therapist and client. Amid this diversity, a common thread of fostering learning and facilitating therapeutic progress emerges, with a recognition of the client's active engagement and empowerment. The process of integrating BSH into psychotherapy is contingent upon the treatment approach, necessitating nuanced considerations, yet grounded in the shared commitment to advancing the client's well-being and progress towards treatment goals.

## Empirical Research on the Relations Between BSH and Outcome

Numerous studies have extensively investigated the relationship between BSH and treatment outcomes, and their findings have been synthesized in multiple meta-analyses and reviews (for a recent comprehensive overview, refer to Ryum et al., 2023b). The majority of earlier publications have focused on three primary areas: (a) assessing the causal impact of homework on treatment outcomes by comparing interventions with and without homework (Kazantzis et al., 2000; Kazantzis, Whittington, et al., 2010); (b) examining the correlation between client compliance with homework and treatment outcomes (Kazantzis, Whittington, et al., 2010; Kazantzis et al., 2016; Mausbach et al., 2010); and (c) investigating therapist skills and competence in assigning homework and their connection to engagement with BSH and treatment outcomes (therapist behaviors), see special issue in *Cognitive Therapy and Research* (Vol. 45, No. 2).

Causal effects analysis has reported a medium effect size (*d* = .53) (Kazantzis et al., 2000; Kazantzis, Whittington, et al., 2010), while a significant linear association (*r* = .26) has been identified between client compliance with homework and treatment outcomes (Mausbach et al., 2010; see also Kazantzis, Whittington, et al., 2010). Recent meta-analyses have further validated these findings concerning both the quantity and quality of homework engagement in relation to treatment outcomes (Kazantzis et al., 2016), including a specific examination of obsessive-compulsive disorder (Wheaton & Chen, 2021). Collectively, these findings underscore that interventions incorporating BSH yield superior outcomes in contrast to those lacking BSH, and that greater client engagement with homework corresponds to better treatment results.

More recent research has progressively highlighted the pivotal role of therapists' skills and competence in integrating BSH to facilitate client engagement with homework (as explored in research question C above). For instance, investigations have revealed that therapist competence in homework review (Bryant et al., 1999; Weck et al., 2013), as well as homework selection and planning (Conklin et al., 2018; Jungbluth & Shirk, 2013; Ryum et al., 2010), correlates with treatment outcomes. Notably, these effects remain robust even when accounting for confounding variables like the therapeutic alliance (McEvoy et al., 2023; Ryum et al., 2022), and studies have also incorporated client feedback in studies of therapist competence in using homework (Hildebrand-Burke et al., 2023; Yew et al., 2021). These findings indicate that the positive impact of BSH extends beyond mere client compliance, emphasizing therapists' pivotal role in facilitating client engagement with homework. Furthermore, a recent review conducted as part of the interorganizational Task Force on Psychotherapy Skills and Methods That Work (Hill & Norcross, 2023) comprehensively summarized findings on therapist behaviors affecting immediate (in-session) and intermediate (session-to-session) outcomes of BSH. The review observed favorable effects on intermediate outcomes, while results for immediate outcomes were mixed and generally neutral (Ryum et al., 2023b). The Task Force concluded that BSH demonstrates efficacy for ultimate treatment outcomes and likely effectiveness for intermediate outcomes (Hill & Norcross, 2023).

However, amidst largely positive results, research has also illuminated challenges that clients may encounter with BSH, stemming from practical and emotional factors, which could impede therapeutic progress and potentially lead to premature discontinuation. Some clients may exhibit adverse reactions to the term “homework” due to its educational connotations, invoking feelings of apprehension linked to evaluation, control, or failure (Fehm & Kazantzis, 2004; Kazantzis, Arntz, et al., 2010). Consequently, the use of this term in clinical practice is not recommended (Kazantzis, MacEwan, et al., 2005), and an alternative, “action plan,” has been proposed within CBT (Kazantzis & Miller, 2022). Additionally, BSH may induce pressure, anxiety, resistance, or exacerbate mood-related issues for various reasons (e.g., lack of comprehension regarding homework rationale, overly demanding assignments due to symptom severity), with some clients identifying it as the foremost challenge in treatment. Consequently, therapists should anticipate potential obstacles, maintain receptivity to client feedback, and establish a collaborative atmosphere where engagement with BSH is discussed through Socratic dialogue and monitored via feedback on session-relevant aspects (Kazantzis et al., 2017).

It should also be recognized that (persistent) non-engagement with BSH may be a way for the client to oppose treatment and/ or the therapist (Okamoto et al., 2019; Okamoto & Kazantzis, 2021; Safran & Muran, 2000; Sijercic et al., 2016). Strong needs for dominance or attachment may be evoked in certain clients, within a therapeutic relationship characterized by giving and receiving help and care, and client non-engagement with BSH may therefore sometimes signal a rupture in the therapeutic alliance (Kazantzis et al., 2023). Although there is little conclusive research in this area, we may speculate if certain client populations are at a higher risk for non-engagement with BSH, for example, as for clients presenting with personality disorders or severe eating disorders. However, in general, we caution against “blaming the client”, and rather suggest that therapists should examine their own contribution in the process when accounting for client’s non-engagement with BSH.

Notably, empirical investigations and reviews of BSH have mainly been conducted within cognitive and behavioral treatment frameworks, with rare exceptions emerging from psychodynamic (Hilsenroth & Slavin, 2008; Nelson & Castonguay, 2017; Owen et al., 2012) and humanistic-experiential approaches. While research underscores BSH's clinical relevance for training and practice, as we will explore in the subsequent section, the generalizability of these findings to alternative treatment methodologies remains uncertain.

## Clinical Practice and Training

Drawing upon theoretical writings and empirical research spanning five decades, our current understanding of the factors that can either facilitate or hinder the effective integration of homework into psychotherapy has become more comprehensive. These factors hold significant relevance for clinical training and practice. A comprehensive practical guide for the utilization of homework already exists in CBT (Kazantzis, MacEwan, et al., 2005). Additionally, an updated and comprehensive model was recently published (Kazantzis & Miller, 2022), which could provide valuable insights for integrating BSH into other therapeutic approaches. In the following sections, we delve into strategies that therapists can employ to enhance client engagement with BSH and discuss how clinical training and supervision can bolster therapist skills and competence in implementing BSH.

### Clinical Practice

While the term "homework" is commonly associated with specific tasks assigned to clients, it also encompasses an in-session process reliant on a collaborative client-therapist relationship. Therapist behaviors play a crucial role in helping clients establish realistic expectations about the role of BSH, fostering engagement with homework, and facilitating symptom improvement. Drawing from practical guides and empirical research, therapists should: (a) collaboratively design, plan, and review BSH in alignment with the client's goals and values; (b) link BSH with takeaways from the session; (c) provide a compelling rationale for homework; (d) address potential challenges and barriers to task engagement; (e) offer a written BSH summary including instructions and rationale; (f) incorporate client feedback when selecting, planning, and reviewing BSH; and (g) remain responsive to the evolving needs and context of the client (Kazantzis, MacEwan, et al., 2005; Kazantzis & Miller, 2022; Ryum et al., 2023a, 2023b). Feedback here refers primarily to the therapist eliciting reactions and input from the client in the on-going therapeutic dialogue (e.g., beliefs about BSH; barriers to engagement; reactions to in-session practice; degree of skill acquisition or mastery), and not to more formal methods such as “routine outcome monitoring” (Lambert & Shimokawa, 2011).

Although “compliance” or “adherence” have historically been associated with BSH, the concept of “engagement” has been proposed as a more meaningful construct for research and clinical practice (Kazantzis & Miller, 2022). Beyond merely measuring completed BSH tasks, a comprehensive assessment of client engagement should encompass potential practical obstacles, task-related difficulties (or perceived difficulties), activation of personal beliefs, and associated emotional distress (Kazantzis, MacEwan, et al., 2005). For instance, a client might view a homework task as “too challenging” or “irrelevant,” leading to negative beliefs about themselves or the therapeutic process. While tracking homework completion remains informative (as compliance is linked to improvement), therapists should also explore client beliefs about BSH benefits, perceived progress contribution, skill acquisition, and encountered obstacles. The Homework Rating Scale-Revised (HRS-II; Kazantzis, Deane, et al., 2005, available from www.cbtru.com) stands as a validated tool towards these goals, and is suitable for research, clinical training, and practice. Regardless of the specific task, BSH should be framed as an opportunity for learning, even when tasks don't proceed as planned.

Therapists might harbor assumptions that hinder the integration of BSH into therapy (Bunnell et al., 2021). Negative attitudes, such as viewing homework as exclusively for distressed clients or fearing it might overstructure the client, should be treated as hypotheses rather than established facts. Integrating BSH should entail Socratic dialogue and feedback solicitation from clients. Moreover, therapists' interpersonal styles or core schemas could impede effective BSH integration, manifesting as “demanding standards” or “excessive self-sacrifice” (Haarhoff & Kazantzis, 2007; Kazantzis et al., 2017).

### Clinical Training

Although research supports the efficacy of BSH for treatment outcomes, empirical investigations into how clinical training and supervision enhance therapists' acquisition and proficient use of BSH are lacking. This gap mirrors the broader dearth of evidence-based training research in the field (Callahan & Watkins, 2018), and the need for future research utilizing controlled, longitudinal designs (see Brattland et al., 2022). We propose recommendations for clinical training based on practice guidelines and insights from process-outcome research (Kazantzis & Miller, 2022; Ryum et al., 2023a, 2023b).

While reading materials and didactic courses grant trainee therapists a conceptual understanding of BSH integration, hands-on practical experiences hold even greater importance. Clinical work necessitates practical skills and competencies, and direct engagement with BSH is pivotal for novice and seasoned therapists alike (see Kazantzis et al., 2017 for competence frameworks for collaborative work in CBT). Competent and skillful BSH utilization hinges on the therapeutic approach, case conceptualization, contextual factors, and in-session dynamics. This complex clinical method defies full standardization but should be informed by practical guidelines. Thus, learning “what works” with various clients and effectively addressing resistance or difficulties requires firsthand experience. Although evaluating how well a therapist sets agendas is relatively straightforward, gauging skilled BSH implementation demands more nuanced expertise.

Role-plays and deliberate practice are effective tools for honing BSH skills in early therapists. Developing standardized stimuli-clips depicting challenging integration scenarios could also enhance training. Nonetheless, supervision of sessions with actual clients, whether through video recordings or direct observation, remains the optimal method for therapists to receive constructive feedback on BSH application. This process can be reinforced using validated measures of therapist behaviors pertinent to BSH, such as the Homework Adherence and Competence Scale (HAACS; Kazantzis, Wedge, et al., 2005, available from www.cbtru.com) or the Homework-Specific Therapist Behaviors Scale (HSTBS; Conklin et al., 2018). These tools aid trainees in identifying and addressing skill gaps related to task selection, planning, review, and overcoming obstacles, and could be used in supervision. Complementing this, a validated measure of client engagement and beliefs, like the HRS-II, could provide insights into client perspectives on BSH, and suggest areas that warrant further attention, exploration, and discussion between therapist and client (e.g., negative beliefs about a specific task; lack of comprehension, rationale, or specificity, etc.), although the scale does not have specific cut-offs.

## Conclusions

There has been more research on the use of BSH in CBT compared to that of other therapist interventions/ methods, and sufficient empirical evidence to consider BSH a demonstrable efficacious method for ultimate treatment outcomes. While empirical support is lacking for other treatment approaches, there is diversity in how BSH may be integrated into clinical work within and across treatment approaches, and we propose that BSH should be considered a transtheoretical method with heuristic value also for psychodynamic and humanistic-experiential therapies. For example, BSH may facilitate the treatment process and outcome by promoting experiential awareness, insight, or the discovery of new meaning; behavior change and the acquisition of new adaptive skills; the generalization of new learning from the counseling room and into the everyday-life of the client; and a sense of agency and confidence in clients that they may play an active role in their own change-process.

Furthermore, empirical evidence highlights specific therapist behaviors linked to skillful BSH utilization, such as presenting a compelling rationale, collaborative planning, addressing challenges, and assigning personalized tasks. These behaviors should be central in clinical practice and training. However, empirical research on how to enhance therapist competence and skill in BSH remains scarce, urging clinicians and researchers to prioritize this vital avenue of inquiry in the future.
